# Domains and Measurements of Healthy Aging in Epidemiological Studies: A Review

**DOI:** 10.1093/geront/gny029

**Published:** 2018-04-20

**Authors:** Wentian Lu, Hynek Pikhart, Amanda Sacker

**Affiliations:** Research Department of Epidemiology and Public Health, Institute of Epidemiology and Health Care, University College London, United Kingdom

**Keywords:** Domain, Measurement, Healthy Aging

## Abstract

**Purpose of the Study:**

Few studies have recommended the essential domains of healthy aging and their relevant measurement to assess healthy aging comprehensively. This review is to fill the gap, by conducting a literature review of domains and measures of healthy aging in epidemiological studies.

**Design and Methods:**

A literature search was conducted up to March 31, 2017, supplemented by a search of references in all relevant articles in English. We made a final selection of 50 studies across 23 countries or regions.

**Results:**

Nineteen studies applied Rowe and Kahn’s three standards to assess healthy aging. Thirty-seven studies measured physical capabilities mainly by (instrumental) activities of daily living. Cognitive functions were included in 33 studies. Nineteen of them applied Mini-Mental State Examination (MMSE). Twenty-six studies considered metabolic and physiological health, but they mainly asked the self-reported absence of diseases. Twenty-four studies assessed psychological well-being by employing diverse scales. Questions about participation in social activities were mainly asked to measure social well-being in 22 studies. Sixteen studies considered individuals’ general health status, which was mainly measured by self-rated health. Security questions were asked in five studies. Health behaviors were taken into account by three studies. Fifteen studies either applied SF-12/36 or developed health indices to assess healthy aging.

**Implications:**

This review summarizes detailed scales or methods that have been used to assess healthy aging in previous epidemiological studies. It also discusses and recommends the essential domains of healthy aging, and the relevant instruments for further epidemiological research to use in the assessment of healthy aging.

In 44 BCE, the Roman philosopher Cicero praised healthy aging in his essay “On Old Age”. He said that old age “is respectable as long as it asserts itself, maintains it rights, is subservient to no one”, indicating that old age is not a phase of decline and loss, but an opportunity for positive changes in later life (Cicero, 44BC/1951, p.140). In the past decades, healthy aging has become a popular topic in many countries. It refers to the process of optimizing opportunities for health, participation, and security, to enhance quality of life as people age (World Health Organization, 2002). “Healthy ageing” is often used interchangeably with terms such as “active”, “successful”, or “productive ageing”. We prefer the term “healthy ageing”, because the World Health Organization (WHO) defines “health” as including not only physical and mental health, but also social well-being (World Health Organization, 2006).

In 1987, Rowe and Kahn defined “successful ageing” on the basis of a psychosocial model. They were responding to the fact that, at that time, many gerontologists only emphasized the role of chronological age in determining individuals’ health, concentrating on average age-related losses across different age groups, and neglecting the substantial heterogeneity of individuals’ health conditions within each age group (Rowe & Kahn, 1987). This heterogeneity is determined by both intrinsic and extrinsic factors of aging. Intrinsic factors are physiological factors such as carbohydrate metabolism, bone density or cognitive function; extrinsic factors are psychosocial factors such as autonomy, control or social support. Rowe and Kahn distinguish between “usual” and “successful” aging. Usual aging is age-intrinsic, nonpathological but high-risk, and focuses on physiological functions and the normal decline of functioning with increasing age. Successful aging is low-risk but high-functioning and implies that extrinsic as well as intrinsic factors play important roles in maintaining individuals’ health within each age group; that is, aging characteristics are age-related rather than age-dependent (Rowe & Kahn, 1987). In 1997, Rowe and Kahn defined successful aging according to three standards: “low probability of disease and disease-related disability and related risk factors”; “high cognitive and physical functional capacity”; and “active engagement with life” (Rowe & Kahn, 1997, p.433). In 2015, Rowe and Kahn suggested adding societal-level principles to evaluate successful aging: more opportunities for employment, voluntary work and social activities, which create new rules and responsibilities for the older adults; trust in older people, because they have accrued knowledge and have a heightened capacity for problem-solving; and investment in training and education for the older adults, rather than their exclusion due to their chronological age (Rowe & Kahn, 2015).

Baltes and Baltes proposed the “selective optimisation with compensation (SOC)” theory of successful aging in 1990. Due to aging loss, individuals may experience restrictions in various functional capabilities, such as in cognitive, emotional, or physical domains. “Selection” can be treated as an adaptive procedure. Individuals may prioritize capabilities in other, new, or transformed domains, and set new life goals, due to environmental demands and their own motivations, skills, and natural capacities. “Optimisation” reflects individuals’ behaviors. People will take advantage of their remaining functions and maximize their chosen life courses, both qualitatively and quantitatively. “Compensation” can be mental and/or technological. When individuals are dealing with situations or goals with insufficient internal capabilities, they can take advantage of external compensatory strategies to cope with internal incapacities, for example, using a hearing aid for hearing loss (Baltes & Baltes, 1990).

In 2014, Kuh and colleagues defined healthy biological aging according three principles: “survival to old age”; “delay in the onset of chronic diseases and disabilities”; and “optimal functioning for the maximal time period” (Kuh, Richards, Cooper, Hardy, & Ben-Shlomo, 2014, p.7). They also suggested that continued social participation, such as through voluntary or paid work, physical activities, or keeping in touch with friends or relatives, is important for the older adults to have an active and meaningful later life, because the social environment that we inhabit across our lives determines the aging process (Kuh et al., 2014).

WHO in 2002 defined active aging as “the process of optimising opportunities for health, participation and security in order to enhance the quality of life as people age” (World Health Organization, 2002, p.12). This definition highlighted the importance of the social environment for the achievement of active aging. In 2015, the WHO used the term “healthy ageing” and defined it as “the process of developing and maintaining the functional ability that enables well-being in older age” (World Health Organization, 2015b). Here, four elements of healthy aging were discussed: functional abilities (health-related attributes that allow people to do what they have reason to value); intrinsic capacities (all the physical and mental capabilities that an individual can draw on); environments (all the factors in the extrinsic world that form the context of a person’s life); and well-being (happiness, security, and fulfilment) (World Health Organization, 2015b). Our fixed personal characteristics (e.g., gender or ethnicity), social norms (e.g., occupation, education, wealth, or social security), and other factors (e.g., smoking, drinking, deprivation, or air pollution) across our life span can affect later health characteristics such as physiological risk factors, diseases, injuries, and broader geriatric syndromes. The cumulative effects of these health characteristics determine one’s intrinsic capacity. Intrinsic capacity and its interaction with the environment determine the functional ability of an individual, and consequently governs the attainment of well-being (World Health Organization, 2015b).

The theory of healthy biological aging and Rowe and Kahn’s theory provide clear standards for researchers to measure healthy aging, whereas Baltes and Baltes’s theory and the WHO concepts tend to introduce disciplines that should be followed when setting public health strategies to achieve healthy aging in different cultural settings. At present, a consensus definition of healthy aging has not been reached.

WHO proposed a public health framework for healthy aging across the life course, which involved developing strategies for health services, long-term care, and environments (World Health Organization, 2015b). But, this report also suggested that before shaping policies, a quantitative assessment of healthy aging to help identify older people’s health and needs are essential (World Health Organization, 2015b). Previous studies have explored the extent of healthy aging based on theories mentioned previously. Previous reviews of healthy aging have summarized different definitions of healthy aging (Bowling & Dieppe, 2005; Cosco, Prina, Perales, Stephan, & Brayne, 2014). One of them listed the specific methods that had been used to measure successful aging, but it mainly discussed the variability in the prevalence of successful agers and the heterogeneity in the sampling and measurements of successful aging (Depp & Jeste, 2006). To our knowledge, no research has followed the WHO recommendation of identifying the essential domains of healthy aging, and made suggestions as to the choice of methods or scales to measure each domain. However, grounded in theories of healthy aging, it is important to fill the gap, since choosing quality measures and a reasonable range of indicators of healthy aging, will help monitor trends in healthy life expectancy and support a country’s aspirations to achieve universal health coverage (World Health Organization, 2015a).

## Methods

### Search Strategy

We searched the PubMed database up to March 31, 2017. All peer-reviewed articles published worldwide before that date in English were eligible for inclusion. The keywords were “healthy ageing”, “measurement”, and several related terms: “successful ageing”, “productive ageing”, “active ageing”, “ageing phenotype”, “assessment”, “evaluation”, and “definition”. These phrases were used with both “ageing” and “aging” spelling conventions. Equations were linked with both “or” and “and”.

### Inclusion and Exclusion Criteria

The articles were epidemiological studies. The main outcome or exposure was healthy, successful, positive, or active aging. Theoretical definitions provided in the absence of detailed measurements of healthy aging were excluded. Studies in genotyping, clinical animal trials and cell tests, and studies that measured healthy aging by using a single subjective question (e.g., “how do you view healthy ageing?”), were excluded.

A standardized protocol was employed to evaluate the quality of each study (Boyle, 1998). Four questions were asked of each article: Does the study design yield a representative sample of the defined target population? Were the measures of healthy aging reliable and valid? Were features of sampling design accounted for in the analysis? Did they report results with confidence intervals?

**Figure 1.**
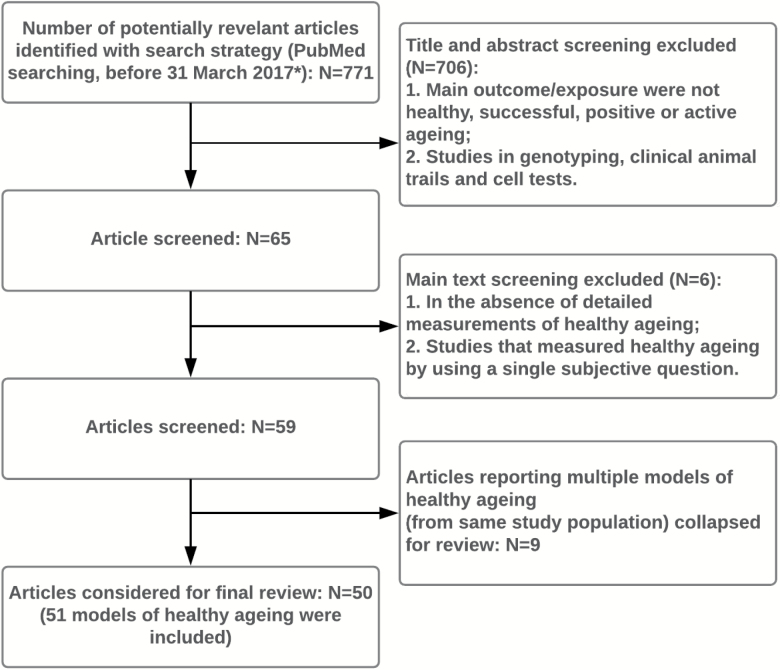
Screening process for studies’ inclusion. *Searching Syntax: ((healthy ageing[Title/Abstract]) OR (successful ageing[Title/Abstract]) OR (active ageing[Title/Abstract]) OR (productive ageing[Title/Abstract])) AND ((measurement[Title/Abstract]) OR (assessment[Title/Abstract]) OR (evaluation[Title/Abstract]) OR (definition[Title/Abstract])) AND (“2017/03/31”[CDAT]: “3000”[CDAT]); ((healthy aging[Title/Abstract]) OR (successful aging[Title/Abstract]) OR (active aging[Title/Abstract]) OR (productive aging[Title/Abstract])) AND ((measurement[Title/Abstract]) OR (assessment[Title/Abstract]) OR (evaluation[Title/Abstract]) OR (definition[Title/Abstract])) AND (“2017/03/31”[CDAT]: “3000”[CDAT]; ((aging phenotype[Title/Abstract]) OR (ageing phenotype[Title/Abstract])) AND (“2017/03/31”[CDAT]; “3000”[CDAT]; ((healthy aging phenotype[Title/Abstract]) OR (healthy ageing phenotype[Title/Abstract])) AND (“2017/03/31”[CDAT]: “3000”[CDAT].

The detailed screening process is presented in Figure 1.

### Defined Domains of Healthy Aging

Lara and colleagues developed five fundamental domains of a healthy aging phenotype in lifestyle-based intervention studies (Lara et al., 2013): physiological and metabolic health; physical capability; cognitive function; social well-being; and psychological well-being. Our review applied these five domains. However, components of healthy aging are complicated due to the various definitions of healthy aging. A few studies in our review also included other components to assess healthy aging. Therefore, we created additional domains such as general health status (e.g., mortality or self-rated health), security (e.g., income or environmental safety), and health behaviors (e.g., smoking or drinking), to categorize them. The social environment is a societal-level component that has been highlighted by a few researchers. However, no studies in our review measured it quantitatively. Our review therefore mainly focused on discussions of components of healthy aging at the individual level.

## Results

**Figure 2.**
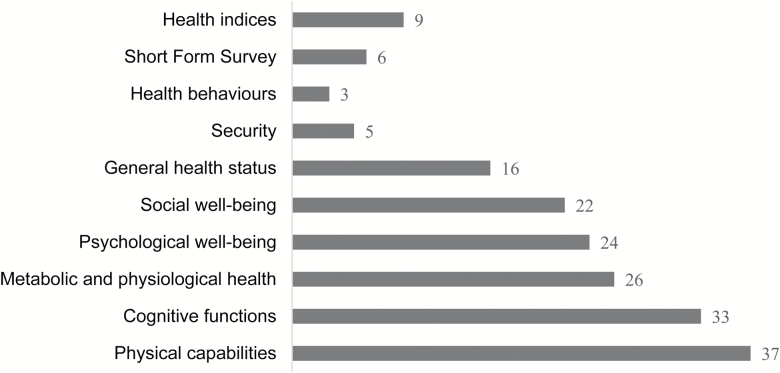
Number of papers measuring each domain.

Fifty articles were selected for analysis. The review covered 23 countries or regions. Most articles mentioned the variability of definitions of healthy aging, but only some of them explicitly clarified the definitions they were using to measure healthy aging. Nineteen studies applied Rowe and Kahn’s three standards (Table 1). Two studies employed the WHO’s active aging model (Table 1). Kuh’s theory of healthy biological aging was used in one study (Table 1). The measurements of healthy aging in each study were multidimensional. Instead of measuring separate domains, several studies used the Short Form Survey 12/36-Item (SF-12/36) or developed health indices to measure healthy aging comprehensively. Table 1 lists the different domains of healthy aging measured in each article. Figure 2 summarizes the number of papers measuring each domain in our review.

**Table 1.** Domains of Healthy Aging in Each Article

Countries	Studies	*N*	Phy.^a^	Cog.^a^	Met.^a^	Psy.^a^	Soc.^a^	Gen.^a^	Sec.^a^	Hea.^a^	Oth.^a^
United States	Women’s Health Initiative (Woods et al., 2012)	71,039	✓	✓			✓	✓			✓
	Health and Retirement Study^b^ (McLaughlin, Connell, Heeringa, Li, & Roberts, 2010)	9,236	✓	✓	✓	✓	✓				
	Health and Retirement Study^b^ (McLaughlin, Jette, & Connell, 2012)	9,068	✓	✓	✓						
	Health and Retirement Study^b^ (Mejia, Ryan, Gonzalez, & Smith, 2017)	17,230	✓	✓	✓	✓	✓				
	Nun Study (Tyas, Snowdon, Desrosiers, Riley, & Markesbery, 2007)	636	✓	✓				✓			
	ORANJ BOWL Panel (Pruchno, Wilson- Genderson, Rose, & Cartwright, 2010)	5,688	✓		✓			✓			
	Successful AGing Evaluation Study (Jeste et al., 2013)	1,006		✓	✓	✓		✓			✓
	- (Kozar-Westman, Troutman-Jordan, & Nies, 2013)	200				✓					✓
	The Georgia Centenarian Study^b^ (Baek, Martin, Siegler, Davey, & Poon, 2016)	306	✓	✓			✓	✓			
	Framingham Offspring Study (McCabe et al., 2016)	1,348		✓	✓						
Mainland China	Shanghai Successful Ageing Project (C. Li et al., 2006)	14,000	✓	✓		✓					
	Chinese Longitudinal Healthy Longevity Survey (Gu, Feng, et al., 2016)	11,095	✓	✓		✓		✓			
	2013 Survey on Life and Opinions of Older Adults in Shanghai^b^ (Gu, Brown, & Qiu, 2016)	1,962						✓			✓
	The China Health and Retirement Longitudinal Study^b^ (Liu et al., 2017)	5,667	✓	✓	✓	✓	✓				
Hong Kong, China	The Hong Kong Centenarian Study^b^ (Cheung & Lau, 2016)	120	✓	✓		✓	✓	✓	✓		
Taiwan	- (C. I. Li et al., 2014)	903									✓
United Kingdom	Whitehall II Study (Akbaraly et al., 2013)	3,044	✓	✓	✓	✓					
	British Longitudinal Survey of Ageing^b,e^ (Bowling & Iliffe, 2006)	999	✓		✓		✓		✓		
	Cambridge City over-75 Cohort Study (Cosco, Stephan, Muniz, & Brayne, 2016)	2,610	✓	✓		✓		✓			
	The West of Scotland Twenty-07 Study^b^ (Whitley, Benzeval, & Popham, 2016)	1,733	✓	✓	✓		✓				
	English Longitudinal Study of Ageing (Felix Caballero et al., 2017)	1,906									✓
Canada	International Mobility in Ageing Study^c,d^ (Belanger, Ahmed, Filiatrault, Yu, & Zunzunegui, 2015)	799	✓		✓		✓	✓	✓		
			✓	✓	✓	✓	✓	✓			
	Canadian Community Health Survey: Healthy Ageing^b^ (Meng & D’Arcy, 2014)	8,154	✓	✓	✓		✓				
	Manitoba Follow-up Study (Tate, Swift, & Bayomi, 2013)	2,043									✓
	-^b^ (Wada, Mortenson, & Hurd Clarke, 2016)	320									✓
Mexico	Health, Wellbeing, and Ageing Study^b^ (Arias-Merino, Mendoza-Ruvalcaba, Arias- Merino, Cueva-Contreras, & Vazquez Arias, 2012)	3,116	✓	✓	✓	✓	✓				
	Coyoacan Cohort^b^ (García-Lara, Navarrete-Reyes, Medina-Mendez, Aguilar- Navarro, & Avila-Funes, 2017)	935	✓	✓	✓		✓				
Singapore	Singapore Longitudinal Ageing Study Cohort (Ng, Broekman, Niti, Gwee, & Kua, 2009)	1,281	✓	✓		✓	✓	✓			
	- (Feng & Straughan, 2016)	1,540									✓
Portugal	Portuguese Project on Active Ageing^b^ (Paul, Ribeiro, & Teixeira, 2012)	1,322	✓	✓	✓	✓	✓		✓	✓	
	Oporto Centenarian Study & Beira Interior Centenarian Study (Araujo et al., 2016)	80	✓	✓	✓	✓	✓				
Brazil	GENESIS Project (de Moraes & de Azevedo e Souza, 2005)	400				✓					
	Ageing, Gender, and Quality of Life Study (Campos, Ferreira, Vargas, & Goncalves, 2016)	335	✓	✓	✓			✓		✓	
France	SUpplémentation en Vitamines et Minéraux AntioXydants Study (Germain et al., 2013)	3,005									✓
	SUpplémentation en Vitamines et Minéraux AntioXydants Study^b^ (Assmann et al., 2016)	2,329	✓	✓		✓					✓
Australia	Melbourne Collaborative Cohort Study^b^ (Hodge, English, Giles, & Flicker, 2013b)	5,512	✓		✓	✓					✓
	The Blue Mountains Eye Study^b^ (Gopinath, Flood, Kifley, Louie, & Mitchell, 2016)	3,654	✓	✓	✓	✓					
Netherlands	The Longitudinal Ageing Study Amsterdam^b^ (Kok, Aartsen, Deeg, & Huisman, 2015)	3,107	✓	✓		✓	✓	✓			
	Rotterdam Study (Jaspers et al., 2017)	3,527	✓	✓	✓	✓	✓				
Cross-countries	EU COURAGE Project (Finland, Poland and Spain) (Perales et al., 2014)	7,987	✓	✓	✓	✓	✓		✓	✓	
	Survey of Health, Ageing and Retirement in Europe (Netherlands, Germany, Italy, Spain, Poland and Hungary) (Sowa, Tobiasz- Adamczyk, Topor-Madry, Poscia, & la Milia, 2016)	11,048	✓					✓			
Mediterranean islands	The Mediterranean Islands Study (Tyrovolas et al., 2014)	2,663	✓		✓	✓	✓		✓	✓	
Spain	Octabaix Project (Formiga et al., 2012)	328	✓	✓							
Norway	Nord-Trøndelag Health Study (Bosnes et al., 2017)	5,773	✓	✓	✓	✓	✓				
Germany	- (Wahl et al., 2013)	450	✓	✓	✓	✓	✓	✓			
Italy	Italian Multi-centric Studies on Centenarians^b^ (Motta, Bennati, Ferlito, Malaguarnera, & Motta, 2005)	602	✓	✓	✓						
Thailand	- (Manasatchakun et al., 2016)	453									✓
South Korea	- (Byun & Jung, 2016)	262									✓
Japan	Aichi Gerontological Evaluation Study (Hirai, Kondo, & Kawachi, 2012)	22,829	✓	✓				✓			
Nigeria	The Biafran War Generation (Chukwuorji, Nwoke, & Ebere, 2017)	453									✓

^a^Phy. = physical capabilities; Cog. = cognitive function; Met. = metabolic and physiological health; Psy. = psychological well-being; Soc. = social well-being; Gen. = general health status; Sec. = security; Hea. = health behaviors; Oth. = others: Short Form Survey or health indices.

^b^Articles applied Rowe and Kahn’s three standards. ^c^This article compared two models of healthy aging: WHO and psychological models.

^d^Articles applied WHO’s active aging model.

^e^Article applied Kuh’s theory of healthy biological aging.

### Physical Capabilities

Thirty-seven studies measured physical capabilities. Twenty-seven of them used the basic activities of daily living (ADLs) and/or instrumental activities of daily living (IADLs). Objective measures were also applied in several articles, including grip strength ([non-] dominant hand arm(s) [kg]), walking speed (timing an eight-foot or six-meter walking course), balance tests (side-by-side standing, semitandem, and full-tandem standing), chair rise tests (standing up from a chair without using arms on time or in seconds), and other functional limitations such as mobility (walking one or several blocks, climbing one or several steps), large muscle (stooping, kneeling, stooping or squatting, and pulling or pushing big objects), or fine motor skills (picking up items between thumb and finger). Two studies measured the frequency of physical activity. One of them employed the WHO Global Physical Activity Questionnaire (Perales et al., 2014). Two studies considered disability. Perales and colleagues applied the WHO Disability Assessment Schedule version II (WHO-DAS II; Perales et al., 2014), and Whitley and colleagues used the Office of Population Censuses and Surveys Disability Scales (OPCS; Whitley et al., 2016).

### Cognitive Functions

Cognitive functions were included in 33 studies. Nineteen of them used the Mini-Mental State Examination (MMSE). Other scales were also employed, including the Alice Heim 4 Test of General Intelligence, the Wechsler Adult Intelligence Scale-Revised, the Montreal Cognitive Assessment Scale (MoCA), the (Modified) Telephone Interview for Cognitive Status, the Subjective Cognitive Failures Questionnaire, the Canadian Community Health Survey-Healthy Ageing Cognition Module, and the Japanese cognitive impairment standards. Rather than choosing established scales, several studies used separate tests to assess cognitive functions, including processing speed (counting backward), short-term memory (immediate or delayed word recall), working memory (digital spanning forward and backward), verbal fluency (animal naming task), orientation to time (specifying month, date, year, day of week, or season), and self-reported memory.

### Metabolic and Physiological Health

Twenty-six studies assessed metabolic and physiological health. Twenty-two of them asked questions about the self-reported absence of chronic diseases such as cancer, lung disease, diabetes, heart disease, stroke, and others. Two studies tested systolic blood pressure. Self-reported hypertension and cardiovascular risk assessment were also included. Lung function was evaluated using forced expiratory volume (l/m^2^) or peak expiratory flow. One study tested the level of fasting glucose for glucose metabolism. Biomarkers such as C-reactive protein (CRP) and creatinine were assessed for cardiovascular and kidney functions, respectively. Two studies measured body mass index (BMI) for body composition. Another two studies considered body pain. One study asked questions about self-rated vision and audition. This study also considered sleeping problems, as inadequate sleep quality, quantity, and timing have been proven to be associated with some metabolic disorders such as impaired glucose tolerance, insulin resistance, or pancreatic β-cell dysfunction (Briançon-Marjollet et al., 2015).

### Psychological Well-being

Twenty-four studies assessed psychological well-being. Studies used a variety of psychological scales. The Centre for Epidemiological Studies-Depression Scale (CES-D), the Geriatric Depressive Screening Scale (GDS), the 9-Item Patient Health Questionnaire, the WHO Composite International Diagnostic Interview, and the Hospital Anxiety and Depression Scale were used to measure depressive symptoms. Life satisfaction was evaluated using the Life Satisfaction Inventory and the Satisfaction with Life Scale (SWLS). The WHO Quality of Life-Brief/100-item scale and the Flanagan Quality of Life Scale assessed individuals’ quality of life. Our review also found many other scales, including the General Health Questionnaire, the Kessler Psychological Distress Scale, the Connor-Davidson Resilience Scale, the University of California Loneliness Scale, the Tenacious Goal Pursuit and Flexible Goal Adjustment Scales, the Environmental Mastery Scale, the Positive and Negative Affect Schedule, the Life Orientation Test-Revised, and the Purpose in Life Test. Additionally, rather than using established scales, some studies asked separate questions about overall life satisfaction, self-rated quality of life, personality, extroversion, openness, happiness and loneliness, maintenance of interest, absence of loneliness and optimism, control and coping, and self-rated mood status.

### Social Well-being

Twenty-two studies measured social well-being. Seventeen of them asked questions about participation in social activities or meetings, such as shopping, leisure activities, voluntary work, childcare, or religious activities. Other questions about social networks of family, friends, and neighbors were found, including about social contact with friends and family, living in company with relatives or friends, and having social chats or activities with neighbors or friends. Several studies measured emotional support, by asking whether participants had family or friends to confide in if they needed emotional support or whether they were being helped/supported in life. One study considered instrumental support by asking whether they helped others with daily tasks in and around the house. Participants’ marital status, paid work status, and subjective autonomy were also investigated. Four scales were used, including the Lubben Social Network Scale, the Classic Circle-Diagram Method, the De Jong-Gierveld Loneliness Scale, and the Oslo 3 Support Scale.

### General Health Status

Sixteen studies asked questions about individuals’ general health. Eleven of them asked for individuals’ self-rated health using a Likert scale (ranging from poorest to excellent). Four studies asked about participants’ subjective feelings regarding successful aging (e.g., how successfully they have aged). One study used mortality to predict healthy aging.

### Security

Socioeconomic security and environmental safety were investigated in five studies. Socioeconomic security was measured by investigating participants’ income or self-perceived financial status or educational attainment. Environmental safety was measured by asking participants whether they felt safe when they were walking in their neighborhood, or to rate facilities and problems in one area.

### Health Behaviors

Smoking, drinking, and/or medication intake were measured in three studies. One study applied the Mediterranean Diet Score to assess nutritional intake.

### Short Form Survey and Health Indices

Rather than measuring separate domains of healthy aging, 15 studies either developed health indices or applied the SF-12/36 to assess healthy aging comprehensively.

Table 2 presents measurements used by the reviewed studies in each domain of healthy aging. Supplementary Table S1 briefly introduces all the established scales (including references) in our review.

**Table 2.** Measurements Used by the Reviewed Studies in Each Domain of Healthy Aging

Dimensions	Methods	Studies
Physical capabilities	Basic/Instrumental Activities of Daily Livings	Akbaraly et al., 2013; Araujo et al., 2016; Arias-Merino et al., 2012; Assmann et al., 2016; Baek et al., 2016; Belanger et al., 2015; Bosnes et al., 2017; Bowling & Iliffe, 2006; Campos et al., 2016; Cheung & Lau, 2016; Cosco et al., 2016; Formiga et al., 2011; García-Lara et al., 2017; Gu et al., 2016; Hodge et al., 2013; Jaspers et al., 2017; Li et al., 2006; Liu et al., 2017; McLaughlin et al., 2010, 2012; Meng & D’Arcy, 2014; Motta et al., 2005; Ng et al., 2009; Paul et al., 2012; Tyas et al., 2007; Wahl et al., 2013; Woods et al., 2012
	Grip strength	Paul et al., 2012; Whitley et al., 2016; Woods et al., 2012
	Walking speed	Akbaraly et al., 2013; Belanger et al., 2015; Woods et al., 2012
	Balance tests	Belanger et al., 2015
	Chair rise tests	Belanger et al., 2015; Woods et al., 2012
	Other functional limitations	Arias-Merino et al., 2012; Campos et al., 2016; García-Lara et al., 2017; Kok et al., 2015; Liu et al., 2017; McLaughlin et al., 2010, 2012; Mejía et al., 2017; Pruchno, Wilson-Genderson, & Cartwright, 2010
	Physical activity	Perales et al., 2014; Tyrovolas et al., 2014
	Disability	Perales et al., 2014; Whitley et al., 2016
Cognitive functions	Mini-Mental State Examination	Araujo et al., 2016; Arias-Merino et al., 2012; Assmann et al., 2016; Baek et al., 2016; Campos et al., 2016; Cheung & Lau, 2016; Cosco et al., 2016; Formiga et al., 2011; García-Lara et al., 2017; Gu et al., 2016; Jaspers et al., 2017; Kok et al., 2015; Li et al., 2006; McCabe et al., 2016; Motta et al., 2005; Ng et al., 2009; Paul et al., 2012; Tyas et al., 2007; Woods et al., 2012
	Alice Heim 4 Test of General Intelligence	Whitley et al., 2016
	Wechsler Adult Intelligence Scale-Revised	Wahl et al., 2013
	Montreal Cognitive Assessment Scale	Belanger et al., 2015
	Modified Telephone Interview for Cognitive Status	Jeste et al., 2013; Liu et al., 2017
	Subjective Cognitive Failures Questionnaire	Jeste et al., 2013
	Canadian Community Health Survey-Healthy Ageing Cognition Module	Meng & D’Arcy, 2014
	Japanese cognitive impairment standards	Hirai et al., 2012
	Processing speed	McLaughlin et al., 2010, 2012; Wahl et al., 2013
	Short-term memory	McLaughlin et al., 2010, 2012; Mejía et al., 2017; Perales et al., 2014; Tyas et al., 2007
	Working memory	Akbaraly et al., 2013; Assmann et al., 2016; Perales et al., 2014
	Verbal fluency	Akbaraly et al., 2013; Assmann et al., 2016; Perales et al., 2014
	Orientation to time	McLaughlin et al., 2010, 2012
	Self-reported memory	Bosnes et al., 2017
Metabolic and Physiological health	Self-reported absence of chronic diseases	Akbaraly et al., 2013; Araujo et al., 2016; Arias-Merino et al., 2012; Belanger et al., 2015; Bosnes et al., 2017; Bowling & Iliffe, 2006; Campos et al., 2016; García-Lara et al., 2017; Gopinath et al., 2016; Hodge et al., 2013; Jaspers et al., 2017; Liu et al., 2017; McLaughlin et al., 2010, 2012; Mejía et al., 2017; Meng & D’Arcy, 2014; Motta et al., 2005; Perales et al., 2014; Pruchno, Wilson-Genderson, & Cartwright, 2010; Wahl et al., 2013; Whitley et al., 2016
	Systolic blood pressure	Akbaraly et al., 2013; McCabe et al., 2016
	Self-reported hypertension	Gopinath et al., 2016; McLaughlin et al., 2012
	Cardiovascular risk assessment	Tyrovolas et al., 2014
	Lung function	Forced expiratory volume Akbaraly et al., 2013; McCabe et al., 2016
		Peak expiratory flow Paul et al., 2012
	Glucose metabolism	McCabe et al., 2016
	Biomarkers	McCabe et al., 2016
	BMI	McLaughlin et al., 2012; Tyrovolas et al., 2014
	Body pain	Jaspers et al., 2017; Pruchno et al., 2010
	Self-rated vision and audition	Paul et al., 2012
	Sleeping problems	Paul et al., 2012
Psychological well-being	Centre for Epidemiological Studies-Depression Scale	Assmann et al., 2016; Belanger et al., 2015; Jaspers et al., 2017; Kok et al., 2015; Kozar-Westman et al., 2013; Liu et al., 2017; McLaughlin et al., 2010; Mejía et al., 2017
	Geriatric Depressive Screening Scale	Araujo et al., 2016; Arias-Merino et al., 2012; Cheung & Lau, 2016; Ng et al., 2009; Tyrovolas et al., 2014
	9-item Patient Health Questionnaire	Jeste et al., 2013
	WHO Composite International Diagnostic Interview	Perales et al., 2014
	Hospital Anxiety and Depression Scale	Bosnes et al., 2017
	Life Satisfaction Inventory	Kozar-Westman et al., 2013; Ng et al., 2009; Paul et al., 2012; Wahl et al., 2013
	Satisfaction with Life Scale	Araujo et al., 2016
	WHO Quality of Life	Brief version Bowling & Iliffe, 2006
		100-item version de Moraes & de Azevedo e Souza, 2005; Paul et al., 2012
	Flanagan Quality of Life Scale	de Moraes & de Azevedo e Souza, 2005
	General Health Questionnaire	Bowling & Iliffe, 2006; Paul et al., 2012
	Kessler Psychological Distress Scale	Hodge et al., 2013
	Connor-Davidson Resilience Scale	Jeste et al., 2013
	University of California Loneliness Scale	Wahl et al., 2013
	Tenacious Goal Pursuit and Flexible Goal Adjustment Scales	Wahl et al., 2013
	Environmental Mastery Scale	Wahl et al., 2013
	Positive and Negative Affect Schedule	Wahl et al., 2013
	Life Orientation Test-Revised	Jeste et al., 2013; Paul et al., 2012
	Purpose in Life Test	Kozar-Westman et al., 2013
	Overall life satisfaction	Gu et al., 2016; Kok et al., 2015
	Self-rated quality of life	Perales et al., 2014
	Personality, extroversion, openness, happiness and loneliness	Paul et al., 2012
	Maintenance of interest and absence of loneliness and optimism	Cosco et al., 2016
	Control and coping	Perales et al., 2014
	Self-rated mood status	Li et al., 2006
Social well-being	Participation in social activities or meetings	Araujo et al., 2016; Baek et al., 2016; Bosnes et al., 2017; Bowling & Iliffe, 2006; Cheung & Lau, 2016; García-Lara et al., 2017; Kok et al., 2015; Liu et al., 2017; Mejía et al., 2017; Ng et al., 2009; Wahl et al., 2013; Whitley et al., 2016; Woods et al., 2012
	Social networks of family, friends, and neighbors	Araujo et al., 2016; Arias-Merino et al., 2012; García-Lara et al., 2017; Kok et al., 2015; McLaughlin et al., 2010; Mejía et al., 2017; Perales et al., 2014; Tyrovolas et al., 2014; Whitley et al., 2016; Woods et al., 2012
	Emotional support	Have family or friends to confide in Cheung & Lau, 2016; Jaspers et al., 2017
		Being helped/supported in life Bowling & Iliffe, 2006
	Instrumental support	Bowling & Iliffe, 2006
	Marital status	Arias-Merino et al., 2012; McLaughlin et al., 2010; Mejía et al., 2017; Whitley et al., 2016
	Paid work status	Arias-Merino et al., 2012; Belanger et al., 2015; Bosnes et al., 2017; García-Lara et al., 2017; McLaughlin et al., 2010; Mejíia et al., 2017; Meng & D’Arcy, 2014
	Subjective autonomy	Wahl et al., 2013
	Lubben Social Network Scale	Paul et al., 2012
	Classic Circle-diagram	Wahl et al., 2013
	De Jong-Gierveld Loneliness Scale	Kok et al., 2015
	Oslo 3 Support Scale	Perales et al., 2014
General health status	Self-rated health	Belanger et al., 2015; Campos et al., 2016; Cheung & Lau, 2016; Cosco et al., 2016; Gu et al., 2016; Kok et al., 2015; Ng et al., 2009; Sowa et al., 2016; Tyas et al., 2007; Wahl et al., 2013; Woods et al., 2012
	Subjective feeling regarding successful aging	Baek et al., 2016; Gu et al., 2016; Jeste et al., 2013; Pruchno et al., 2010
	Mortality	Hirai et al., 2012
Security	Socioeconomic security	Income or financial status Belanger et al., 2015; Bowling & Iliffe, 2006; Cheung & Lau, 2016; Paul et al., 2012; Tyrovolas et al., 2014
		Educational attainment Paul et al., 2012; Tyrovolas et al., 2014
	Environmental safety	Feel safe Perales et al., 2014
		Rate facilities and problems Bowling & Iliffe, 2006
Health behaviors	Smoking, drinking and (or) medication intake	Campos et al., 2016; Paul et al., 2012; Perales et al., 2014
	The Mediterranean Diet Score	Tyrovolas et al., 2014
Short Form Survey and health indices	Health indices	Byun & Jung, 2016; Chukwuorji et al., 2017; Caballero et al., 2017; Feng & Straughan, 2016; Gu et al., 2016; Kozar-Westman et al., 2013; Manasatchakun et al., 2016; Tate et al., 2013; Wada et al., 2016
	Short Form-12/36 Health Survey (SF-12/36)	Assmann et al., 2016; Germain et al., 2013; Hodge et al., 2013; Jeste et al., 2013; Li et al., 2014; Woods et al., 2012

## Discussion

### From Biological Aging to the Psychosocial Model and Resilience

Kuh’s theory of healthy biological aging mostly focused on longevity, the absence of diagnosed chronic diseases, and the minimization of functional deterioration and disability. Rowe and Kahn’s theory involved psychosocial components of healthy aging. However, both held the opinion that social engagement and mental capacities are as important as biological factors, since aging characteristics are age-related rather than age-dependent. Psychological and social well-being are measured in order to examine the effects of self-efficacy, social roles, and social support on functional well-being. Older people have fewer friends and family and are more likely to feel isolated and lonely; but they benefit more than younger generations from participation in social activities and interactions with others, which contributes to better emotional regulation and greater well-being (Stafford, McMunn, Zaninotto, & Nazroo, 2011).

Rowe and Kahn also highlighted the importance of social structure, suggesting that more social opportunities should be provided for older people. The WHO 2012 active aging model similarly emphasized the importance of providing more social opportunities for the older adults. The WHO 2015 healthy aging model used the term “environment” to cover all components of healthy aging in the external world at different levels, such as the neighborhood environment, people’s relationships, and social policies and services. In future research, it is essential to consider environmental indicators of healthy aging, because the interaction between a person and their social environment can explain most of the variability in intrinsic capacities in older age (Brooks-Wilson, 2013).

Some researchers have criticized Rowe and Kahn’s three standards as providing a “perfect” definition, because Rowe and Kahn excluded older people with any evidence of incapacity and retained only a small “elite” group of the older adults (Lowry, Vallejo, & Studenski, 2012). Several studies in this review likewise classified healthy agers by only categorizing individuals who were free of any impairment or illness into a healthy aging group (Assmann et al., 2016; Bosnes et al., 2017; Campos, Ferreira, Vargas, & Gonçalves, 2016; García-Lara et al., 2017; Gopinath, Flood, Kifley, Louie & Mitchell, 2016; Liu et al., 2017; Sowa et al., 2016; Wada, Mortenson, & Hurd Clarke, 2016). According to Baltes and Baltes’s SOC theory, many older people have impairment in one or more domains, but they may still be capable of taking advantage of their remaining capacities and compensating for any losses or limitations. SOC focuses on the importance of resilience, allowing for self-efficacy and growth in the context of increased biological vulnerability and reduced capabilities. Similar to Baltes and Baltes, the WHO 2015 healthy aging model also recognized older people’s ability to maintain and improve a level of functional ability in the face of adversity. It said that seniors might preserve some functional skills without drawing on them at particular points in time, and that these preserved skills could contribute to their resilience. Therefore, when measuring healthy aging, researchers need to consider whether a “disease-free” aging status is achievable, to ensure the classification of healthy agers does not import any selection bias.

### Essential Domains of Healthy Aging

Although previous studies measure healthy aging differently, our review still found similar trends in the demographic, socioeconomic, psychosocial, and behavioral inequalities of healthy aging. For example, men, younger, and married participants tended to be healthier than those who were women, older, and unmarried (Araújo, Ribeiro, Teixeira, & Paúl, 2016; Campos et al., 2016; Cosco et al., 2016; Jaspers et al., 2017; Liu et al., 2017). Older people who had better psychosocial well-being, such as less stress and more family support, could more easily achieve healthy aging than others (Baek, Martin, Siegler, Davey, Poon, 2016; Byun & Jung, 2016; Chukwuorji et al., 2017; Gu, Brown, & Qiu, 2016; Kok, Aartsen, Deeg, & Huisman, 2015). People in more advantaged socioeconomic groups were more likely to have less illness in later life (Caballero et al., 2017; Kok et al., 2015; Liu et al., 2017; Whitley, Benzeval, & Popham, 2016). Those who were nonsmokers, took more physical exercise and had higher nutritional intakes, were also more likely to become healthy agers (Bosnes et al., 2017; Sowa, Tobiasz-Adamczyk, Topor-Madry, Poscia, & la Milia, 2016). The abilities of the different healthy aging models to distinguish inequalities in healthy aging were similar, indicating that the development of a single worldwide metric of healthy aging seems unnecessary.

However, our review shows that the domains of physical capability, cognitive function, metabolic and physiological health, psychological well-being, and social well-being are more frequently used than other domains to assess healthy aging (Figure 2). Furthermore, more instruments were applied to measure them (Table 2). They are important components of healthy aging in Rowe and Kahn’s theory. Previous reviews have also recognized them as important characteristics of healthy aging (Bowling & Dieppe, 2005; Cosco, Prina, Perales, Stephan, & Brayne, 2014; Depp & Jeste, 2006).

Sufficient studies have found an association of these five domains with morbidity or mortality. For example, higher blood pressure, and low-density lipoprotein cholesterol and fasting glucose contribute to adverse cardiometabolic events in older age (Lawlor & Hardy, 2014). A meta-analysis has indicated that weaker grip strength, slower walking speed, and poorer performance in chair rise and standing balance tests in older people are all associated with an increased risk of all-cause mortality (Cooper, Kuh, & Hardy, 2010). Another study among community-dwelling older adults showed that cognitive decline, especially among the young older adults, had a significantly adverse impact on longevity (Bassuk, Wypij, & Berkman, 2000). One study found that social-emotional support, such as receiving verbal encouragement, getting married, or participating in social activities, was positively associated with neuroendocrine function and physical performance among the aging population (Glass, Seeman, Herzog, Kahn, & Berkman, 1995).

Therefore, physical capabilities, cognitive functions, metabolic and physiological health, psychological well-being, and social well-being are identified in our review as essential domains for future epidemiological research to assess healthy aging.

### Measurements in Each Domain of Healthy Aging

Physical capability is the degree to which a person can manage the physical tasks of daily living (Segen, 1992). (I)ADLs were the most frequently used instruments to measure this in our review. One community-based study endorsed the application of (I)ADLs, saying that they can describe a broader range of needs among the older adults (Spector, Katz, Murphy, & Fulton, 1987). Another community-based study also recognized IADLs as a good discriminator of physical incapacities, but expressed concern that items such as food preparation, housekeeping, and laundry were highly relevant to women, resulting in reporting bias among men (Avlund, Schultz-Larsen, & Kreiner, 1993). A hospital-based study preferred to use direct observations of performance, such as grip strength, walking speed, balance, or the chair rise test, to predict physical capabilities, as patients consistently overrated their own abilities in (I)ADLs (Edwards, 1990). Physical activity is difficult to measure accurately, as it comprises work, transport, and entertainment activities. More importantly, when asking participants about physical activities, researchers sometimes used terms such as “exercise” or “fitness” rather than “activity”, but these terms are not interchangeable (Caspersen, Powell, & Christenson, 1985). For the absence of disability, neither WHODAS2.0 nor OPCS measures disabilities in terms of only physical capabilities. Both also consider disability in other respects, such as cognitive disability, or disability in self-care activities or getting along with others (World Health Organization, 2010). Therefore, ADLs and IADLs are recommended for community-based studies to predict physical capabilities. Also, it is better to include direct observations of performance to improve predictability, especially when measuring physical capabilities among men.

Cognitive function is an intellectual process by which one becomes aware of, perceives, or comprehends ideas. It is related to knowledge, attention, memory, judgment, evaluation, and more (Segen, 1992). MMSE was the most frequently applied scale in our review. It is valid for identifying severe cognitive impairment, but it is not sensitive to mild cognitive impairment, and it should not be used as a diagnostic tool to identify early signs of dementia (Simard, 1998; Tombaugh & McIntyre, 1992). One potential reason for this is that the tests of memory and executive functions in MMSE are quite limited. MMSE has no recognition paradigms, vision, personal, or working memory measures, and no tests of the capacity to abstract or judge social situations (Simard, 1998). Researchers compared MMSE with MoCA, indicating that MoCA has more reliable psychometric properties to detect mild cognitive impairment or dementia (Hoops et al., 2009). Researchers have suggested using MMSE along with other measures to enhance the validity of cognitive function evaluations (Simard, 1998). For example, MMSE could be applied together with tests of short-term memory (immediate or delayed word recall), working memory (digital spanning forward and backward), verbal fluency (animal naming task), or orientation to time (specifying month, date, year, day of week, or season). In this review, two studies used MMSE along with tests of short-term memory (Tyas, Snowdon, Desrosiers, Riley, & Markesbery, 2007) or verbal fluency and working memory (Assmann et al., 2016), which may provide more detailed information about participants’ cognitive functions.

In the domain of metabolic and physical health, around 85% articles asked questions about the self-reported absence of diseases. However, little previous research has discussed the validity of these questions. One study suggested that men were more likely to over-report stroke but under-report malignancies; women tended to over-report malignancies and arthritis; and older age was associated with both over- and under-reporting of cardiac diseases, over-reporting of stroke and under-reporting of arthritis (Kriegsman, Penninx, van Eijk, Boeke, & Deeg, 1996). Apart from asking questions about the absence of diseases, one study also added tests for systolic blood pressure, forced expiratory volume (l/m^2^), fasting glucose, CRP, and creatinine to provide more objective results (Akbaraly et al., 2013). Two studies used BMI as a surrogate measure of body fat. However, researchers argued that aging is accompanied by a progressive increase in the ratio between fat and lean body mass, and BMI fails to detect the “conversion” of lean to fat tissue (Prentice & Jebb, 2001). Only two studies measured body pain, sleeping problems, and/or self-reported vision and audition, although these are important indicators of frailty for the aging population (Rockwood et al., 2005). In summary, we cannot deny that the self-reported absence of chronic diseases may involve reporting bias, but it has frequently been used in many previous studies. Objective tests for cardiovascular and lung function, glucose metabolism, sleeping problems, vision, audition, and body pain are able to provide more accurate information on individuals’ metabolic and physical health.

The validity of psychological scales has been confirmed in previous studies. For example, CES-D is capable of distinguishing the severity of depressive symptoms and providing valid information for psychiatric treatment (Radloff, 1977). GDS is internally consistent with the Hamilton Rating Scale for Depression or the Zung Self-Rating Depression Scale and is significantly correlated with Research Diagnostic Criteria for depression (Yesavage et al., 1982). SWLS is correlated moderately to highly with subjective well-being and is suitable for different age groups (Diener, Emmons, Larsen, & Griffin, 1985). However, different studies have used different terminology to specify what they measure, even on the same scale, and items are used interchangeably across studies—for example, “depression” and “depressive symptoms”; “distress” and “disorder”; or “emotional”, “psychological”, and “mental” well-being. None of the screening scales reflects a specified conceptual domain. Researchers failed to explain conceptually what they were attempting to measure. Therefore, it is difficult to conduct comparisons across studies. When researchers are choosing scales to measure psychological well-being, it is necessary to clearly distinguish between the concepts behind the measurements, rather than only focusing on the validity and reliability of scales.

Measurements of social well-being are fuzzy, and there are no clear boundaries between them. Norms and expectations of social well-being vary across different cultures and social classes. The commonest way to avoid these issues is to focus on measuring specific social roles. There is agreement on several social behaviors, such as being involved in the community or paid employment, doing housework, being a parent or spouse, or having leisure activities (McDowell, 2006a). Many studies in our review measured these social behaviors by asking questions about the frequency of engaging in social activities or meetings, helping others, paid work status, and marital status. Recent research also emphasizes the importance of participating in creative activities for healthy aging, suggesting that developing a long-term and substantial interest in a hobby, with the goal of attaining skills, may improve older people’s adaptation to later life (Adams-Price, Nadorff, Morse, Davis, & Stearns, 2018).

Both health indices and the Short Form Health Survey are designed to summarize different aspects of health in an overall score, with the aim of developing health metrics to assess healthy aging comprehensively. Rather than dichotomizing healthy and unhealthy aging, they determine healthy aging on the basis of a continuous rating, which mostly avoids the risk of only recognizing participants with no incapacity as healthy agers. However, all the studies calculated the final score simply by summing each indicator score, potentially resulting in inaccurate assessments of participants’ health, since a participant’s severe illness in one domain will be neglected if the person gets an intermediate sum score due to better health in other domains. Some researchers hold a similar opinion, stating that respondents can attain an intermediate score in many ways, which does not provide interpretable information on health (McDowell, 2006b). However, it is rare that epidemiologists have considered how to calculate the parameters of each health indicator. A recent study developing a mortality risk index allocated parameters for each health indicator based on the beta coefficient that each indicator achieved when predicting mortality (Kobayashi, Jackson, Lee, Wardle, & Steptoe, 2016).

Questions about self-rated health or subjective feelings about successful aging can only provide supplementary information about healthy aging, as they cannot capture specific characteristics. In relation to security and health behaviors, many studies in our review mainly recognized these as social determinants of healthy aging, rather than as components of it. For example, one study indicated that people with higher educational attainment were more likely to achieve healthy aging (Perales et al., 2014). Another study suggested that participants with lower incomes would attain lower healthy aging scores (Manasatchakun et al., 2016). Consuming more fiber-rich food or following nutritional intake guidelines was also proven to be beneficial for healthy aging (Assmann et al., 2016; Germain et al., 2013; Gopinath et al., 2016; Tyrovolas et al., 2014).

## Implications

This review discussed the theoretical development of healthy aging; recommended essential domains of healthy aging; and made suggestions as to the choice of instruments to measure healthy aging. Measures in physical and cognitive functions, as well as psychological and social well-being, were in correspondence with instruments provided in the NIH Toolbox (National Institutes of Health, 2004), an important e-resource which includes comprehensive and validated assessment tools to evaluate neurological and behavioral functions among individuals aged 3–85 years. This review provides convincing guidelines toward the development of well-suited assessments of healthy aging in epidemiological studies. It may also help clinicians select simple, but efficient and understandable health indicators to identify healthy agers in prevention or intervention trials. Moreover, this review may be useful for policy-makers to capture key elements of healthy aging, develop aging policies in social, economic and civic affairs, and optimize opportunities for older people’s health, social participation, and security.

## Limitations

Our review only includes English-language publications. Articles in other languages may introduce more country-specific indicators of healthy aging. For example, a study reported that the dependence on family was a predictor of healthy aging among Singaporeans (Feng & Straughan, 2016). Also, we only searched the PubMed database, which may affect the representativeness of the data. However, our review recommended coherent domains and measurements of healthy aging to those which are provided in the NIH Toolbox. Another limitation is that it is data-driven. We can only report on the available variables in studies, but these may not meet researchers’ full requirements. Our recommendations for measurements of healthy aging can only be developed on the basis of the variables they used. Moreover, although theories of healthy aging suggest avoiding negative attitudes or norms about the older adults, and focusing on measuring well-being rather than losses or limitations, previous studies still assessed healthy aging negatively, as most current methods or scales measure illness or impairment rather than well-being.

## Conclusion

In conclusion, psychosocial components are as important as biological components in healthy aging. Future research needs to consider the environmental indicators of healthy aging. Also, researchers need to think about whether a “disease-free” aging status is achievable in their samples, to avoid selection bias when identifying healthy agers.

The development of a single worldwide metric of healthy aging seems to be unnecessary. However, our review identified physical capabilities, cognitive functions, metabolic and physiological health, psychological well-being, and social well-being as more frequently used domains than others.

ADLs and IADLs are recommended for community-based studies to predict physical capabilities. It is also better to test direct observations of performance such as grip strength, walking speed, balance, and the chair rise test to improve predictability, especially when measuring physical capabilities among men. MMSE is not the most appropriate scale to evaluate cognitive functions, but it can provide a brief cognitive screening test, and has been used in this way in numbers of population-based studies. Its application along with other cognitive tests, especially in memory and executive functions, is recommended. Self-reported absence of chronic diseases may result in reporting bias, but it has been used in many studies. It is better to have objective tests for cardiovascular and lung functions, glucose metabolism, sleeping problems, vision, audition, and body pain. However, BMI may not be an appropriate indicator of body composition for the aging population. When one is choosing scales to measure psychological well-being, rather than only focusing on the validity and reliability of scales, it is more important to clearly distinguish the concepts behind the measurements. Measurements of social well-being are fuzzy, but measurements of specific social roles are common in previous research. When developing health indices or applying SF-12/36 to measure healthy aging, it is recommended that one should consider the parameters of each health indicator, because different indicators may play different roles for individuals in promoting healthy aging.

## Funding

WL was supported by the UCL Overseas Research Scholarship and the China Scholarship Council (File No. 201608060385). AS was supported by the Economic and Social Research Council (Award No. ES-J019119/1 ).

## Conflict of Interest

None reported.

## Supplementary Material

gny029_suppl_Supplementary_Material
