# Supplementary material for: Domains and Measurements of Healthy Aging in Epidemiological Studies: A Review
Source: Gerontologist. 2018 Apr 20;59(4):e294–310. doi: 10.1093/geront/gny029 (PMC6630160; doi:10.1093/geront/gny029)
Supplement: gny029_suppl_Supplementary_Material [file gny029_suppl_supplementary_material.docx]

# **Supplementary Data**

**Table S1. Outline of Established Scales**

| Dimensions | Methods | Outline |
| --- | --- | --- |
| Physical capabilities | Basic Activities of Daily Living | Evaluates individuals’ basic functioning, including self-care tasks, such as walking, dressing, bathing, eating, getting in or out of bed, and using the toilet (Pendleton & Schultz-Krohn, 2008). |
|  | Instrumental Activities of Daily Living | Evaluates individuals’ capability to live independently in a community, including more advanced-level skills in all performance areas, such as using a telephone or computer, managing money, cooking, shopping and taking medication (Pendleton & Schultz-Krohn, 2008). |
|  | WHO Global Physical Activity Questionnaire | A standardised tool to measure physical activity that enables comparisons across culturally diverse populations, collecting information on physical activity in three domains: employment, transport and discretionary time (World Health Organization, 2002). |
|  | WHO Disability Assessment Schedule version II | A generic assessment instrument for health and disability which covers six domains: cognition, mobility, self-care, getting along, life activities and participation (World Health Organization, 2012). |
|  | Office of Population Censuses and Surveys Disability Scales | Thirteen scales that can be summed to give disability dimension scores and a total score. The thirteen scales include items covering locomotor, reaching and stretching, dexterity, personal care, continence, seeing, hearing, communication, behaviour, intellectual function, consciousness, eating/drinking/digestion and disfigurement. (Kelly & Jessop, 1996). |
| Psychological well-being | Centre for Epidemiological Studies-Depression Scale | A self-reported depression scale for the general population, designed for measuring depressed mood, psychological symptoms, well-being and social desirability, which has become the workhorse of depression epidemiology since the 1970s (Radloff, 1977). |
|  | Geriatric Depressive Screening Scale | Thirty items for measuring depressive symptoms, specially designed for rating depression among the elderly, and used extensively in community, acute and long-term care settings since the 1980s (Yesavage et al., 1982). |
|  | 9-item Patient Health Questionnaire | A nine-item depression module from the full questionnaire to make criteria-based diagnoses of depressive disorders and grade the severity of depressive symptoms (Kroenke, Spitzer, & Williams, 2001). |
|  | World Mental Health Survey Initiative version of the WHO Composite International Diagnostic Interview | A screening module developed by WHO to detect mental disorders among the general population, including forty sections that focus on diagnoses, functioning, treatment, risk factors, socio-demographic correlates and methodological factors (Kessler & Ustun, 2004). |
|  | Hospital Anxiety and Depression Scale | A self-reported scale for detecting depression and anxiety in the setting of an hospital medical outpatients clinic (Zigmond & Snaith, 1983). |
|  | Life Satisfaction Inventory | A self-reported questionnaire which tests general feelings of well-being among older people to identify “successful ageing”, measuring five components of life satisfaction: zest, resolution and fortitude, congruence between desired and achieved goals, positive self-concept, and mood tone (Neugarten, Havighurst, & Tobin, 1961). |
|  | WHO Quality of Life | A multidimensional instrument to measure quality of life in psychological and physical health, social relationships and the environment (World Health Organization). |
|  | Flanagan Quality of Life Scale | A fifteen-item instrument that measures five conceptual domains of quality of life: material and physical well-being, relationships with other people, social, community and civic activities, personal development and fulfilment, and recreation (Burckhardt & Anderson, 2003). |
|  | General Health Questionnaire | A screening instrument for common mental disorders, which has frequently been used in busy clinical settings, as well in settings where patients need help to complete the questionnaire. It includes items such as anxiety and depression, social dysfunction and loss of confidence (Sanchez-Lopez Mdel & Dresch, 2008). |
|  | Satisfaction with Life Scale | A self-reported scale designed for an overall judgement of individuals’ life satisfaction (Diener, Emmons, Larsen, & Griffin, 1985). |
|  | Kessler Psychological Distress Scale | A (ten- or six-question) screening scale of psychological distress for the redesigned US National Health Interview Survey, including items such as depressed mood, anhedonia, eating, sleeping, motor agitation, motor retardation, fatigue, worthless guilt, concentration, death, anxiety, worry, motor tension, hypersensitivity, vigilance and positive affect (Kessler et al., 2002). |
|  | Connor-Davidson Resilience Scale | A self-reported scale comprised of twenty-five items to measure resilience – an individual’s ability to thrive despite adversity (Campbell-Sills & Stein, 2007). |
|  | University of California Loneliness Scale | A self-reported twenty-item scale to measure current loneliness and related emotional states (Russell, Peplau, & Ferguson, 1978). |
|  | Tenacious Goal Pursuit and Flexible Goal Adjustment Scales | Self-reported instruments to assess individuals’ dispositional tendency to use assimilative and accommodative coping strategies, regularly used in ageing research (Henselmans et al., 2011). |
|  | Environmental Mastery Scale | An instrument to test whether an individual has a sense of mastery and competence in managing their environment, controls a complex array of external activities, makes efficient use of surrounding opportunities, or can choose or create a context suitable for their personal needs and values (Ryff, 1989). |
|  | Positive and Negative Affect Schedule | Two ten-item mood scales. Descriptors such as attentive, interested, alert, excited, enthusiastic, inspired, proud, determined, strong and active are included in the Positive Affect Scale; distressed, upset, hostile, irritable, scared, afraid, ashamed, guilty, nervous and jittery are included in the Negative Affect Scale (Watson, Clark, & Tellegen, 1988). |
|  | Life Orientation Test-Revised | A revised version of LOT containing twelve items to measure optimism versus pessimism, four worded positively, four worded negatively and four fillers (Scheier, Carver, & Bridges, 1994). |
|  | Purpose in Life Test | An instrument designed to assess perceived meaning and life purpose, including items such as enthusiasm, excitement in living, presence of clear life goals, life being meaningful, newness of each day, wishing for more lives, activity after retirement, life goal completion, good things in life, life lived having been worthwhile, and more (Crumbaugh & Maholick, 1964). |
| Cognitive functions | Mini Mental State Examination | A tool to measure cognitive impairment, consisting of seven categories: orientation to time, orientation to place, registration of three words, attention and calculation, recall of three words, language and visual construction (Tombaugh & McIntyre, 1992). |
|  | Wechsler Adult Intelligence Scale-Revised | A general test of intelligence, consisting of six verbal subtests (information, comprehension, arithmetic, digit span, similarities and vocabulary) and five performance subtests (picture arrangement, picture completion, block design, object assembly, digit symbol) (Wechsler). |
|  | Montreal Cognitive Assessment Scale | A tool to screen patients who present with mild cognitive complaints and usually perform in the normal range on MMSE. It consists of eleven categories: orientation, drawing figures, processing speed, naming objects, memory, recall, attention, vigilance, repetition, verbal fluency and abstraction (Nasreddine et al., 2005). |
|  | Modified Telephone Interview for Cognitive Status | A modified version of TICS to assess cognitive status based on a telephone interview screening, which is modelled after MMSE and composed of orientation, memory, simple attention, working memory, and verbal episodic memory (van den Berg, Ruis, Biessels, Kappelle, & van Zandvoort, 2012). |
|  | Canadian Community Health Survey-Healthy Ageing Cognition Module | A module that includes four cognitive tests: immediate and delayed word recall, animal-naming and the Mental Alternation Test (a simple and practical cognitive assessment tool which involves the timed performance of a sequencing and category switching task (Salib & McCarthy, 2002)) (Meng & D'Arcy, 2014). |
|  | Japanese cognitive impairment standards | An instrument that assesses functional decline or cognitive impairment, and makes a screening judgement based on the opinions of a regular doctor (Hirai, Kondo, & Kawachi, 2012). |
|  | Alice Heim 4 Test of General Intelligence | A test to measure fluid intelligence, composed of sixty-five verbal and mathematical reasoning items of increasing difficulty (Heim, 1970). |
|  | Subjective Cognitive Failures Questionnaire | A questionnaire measuring self-reported failures in perception, memory and motor function (Broadbent, Cooper, FitzGerald, & Parkes, 1982). |
| Social well-being | Lubben Social Network Scale | An instrument to assess social integration and screen for social isolation among older adults, including family and friend networks and interdependent social supports (Lubben, 1988). |
|  | Classic Circle-diagram | An instrument comprising three differently sized circles that individuals use to differentiate the importance of their social networks: circle one includes persons to whom one feels closest emotionally, while circle three includes persons to whom one feels least close, but who are important for other reasons (Kahn & Antonucci, 1980). |
|  | De Jong-Gierveld Loneliness Scale | A unidimensional loneliness scale, consisting of emotional and social subscales including items such as severe deprivation, abandonment, missing companionship, sociability and meaningful relationships (Jong-Gierveld & Kamphuis, 1985). |
|  | Oslo 3 Support Scale | A short questionnaire with questions about the number of close confidants, the sense of concern or interest from other people, and relationships with neighbours (Dalgard et al., 2006). |
| Nutritional intake | The Mediterranean Diet Score | A diet questionnaire that asks questions about the frequency of consumption (servings per month) in eleven food categories, including non-refined cereals, potatoes, fruits, vegetables, legumes, fish, red meat and products, poultry, and full-fat dairy products (Panagiotakos, Pitsavos, & Stefanadis, 2006). |
|  | Short Form Health Survey | A multipurpose short-form health survey with thirty-six questions, including both physical and mental health summary measures, as opposed to surveys that target a specific age, disease or treatment group. Sections are vitality, physical functioning, body pain, general health perceptions, physical role functioning, emotional role functioning, social role functioning and mental health (Ware, 2000). |
